# Higher neonatal growth rate and body condition score at 7 months are predictive factors of obesity in adult female Beagle dogs

**DOI:** 10.1186/s12917-017-0994-7

**Published:** 2017-04-13

**Authors:** Lucie Leclerc, Chantal Thorin, John Flanagan, Vincent Biourge, Samuel Serisier, Patrick Nguyen

**Affiliations:** 1 LUNAM University, Oniris, Nantes-Atlantic College of Veterinary Medicine and Food Science and Engineering, Nutrition and Endocrinology Unit, C.S. 40706, 44307 Nantes Cedex 03, France; 2 LUNAM University, Oniris, Nantes-Atlantic College of Veterinary Medicine and Food Science and Engineering, Animal Physiopathology and Functional Pharmacology, C.S. 40706, 44307 Nantes Cedex 03, France; 3 Royal Canin SAS, 650 avenue de la petite Camargue, 30470 Aimargues, France; 4 Nutrition and Endocrinology Unit, Oniris, Nantes-Atlantic College of Veterinary Medicine and Food Sciences and Engineering, C.S. 40706, 44307 Nantes Cedex 03, France

**Keywords:** Canine, Energy imbalance, Growth, Obesity, Overweight, Predictive factors

## Abstract

**Background:**

The risks during early growth on becoming overweight in adulthood are widely studied in humans. However, early-life predictive factors for canine adult overweight and obesity have not yet been studied.

To identify factors that may help explain the development of overweight and obesity at adulthood in dogs, a longitudinal study of 2 years was conducted in 24 female Beagle dogs of the same age, sexual status, and raised under identical environmental conditions. By means of a hierarchical classification on principal components with the following quantitative values: fat-free mass (FFM), percentage fat mass and pelvic circumference at 2 years of age, three groups of dogs were established and were nominally named: ideal weight (IW, *n* = 9), slightly overweight (OW1, *n* = 6) and overweight (OW2, *n* = 9). With the aim of identifying predictive factors of development of obesity at adulthood parental characteristics, growth pattern, energy balance and plasma factors were analysed by logistic regression analysis.

**Results:**

At 24 months, the group compositions were in line with the body condition scores (BCS 1–9) values of the IW (5 or 6/9), the OW1 (6/9) and the OW2 (7 or 8/9) groups. Logistic regression analysis permitted the identification of neonatal growth rate during the first 2 weeks of life (GR_2W_) and BCS at 7 months as predictors for the development of obesity at adulthood. Seventy percent of dogs with either GR_2W_ >125% or with BCS > 6/9 at 7 months belonged to the OW2 group. Results from energy intake and expenditure, corrected for FFM, showed that there was a greater positive energy imbalance between 7 and 10 months for the OW2, compared to the IW group.

**Conclusion:**

This study expands the understanding of previously reported risk factors for being overweight or obese in dogs, establishing that (i) 15 out of 24 of the studied dogs became overweight and (ii) GR_2W_ and BCS at 7 months of age could be used as predictive factors as overweight adult dogs in the OW2 group had higher values compared the other groups of dogs.

**Electronic supplementary material:**

The online version of this article (doi:10.1186/s12917-017-0994-7) contains supplementary material, which is available to authorized users.

## Background

Around 50% of pet dogs have been reported to be overweight or obese [1–3], which makes these, conditions an important health concern in small animal medicine. Overweight and obese dogs are defined as having excessive adipose tissue that results in a body weight (BW) >15 and >30% above their ideal BW, respectively [4]. The main reason for excess weight gain in healthy dogs is a positive imbalance between energy intake and energy expenditure [4]. Obesity in dogs is associated with decreased quality of life and lifespan [5, 6], as well as with numerous chronic disorders such as osteoarthritis and cardiorespiratory diseases [4]. Some of those obesity-related outcomes can be reversed by restricted energy intake and by increased activity through a weight-loss program [7]. However, only half of the dogs entering such program reach their target body weight [8] and among them, only half succeed in maintaining their optimal body weight over the long term [9]. In the case of obesity in adulthood, both human and animal studies have reported a dysregulation of plasma biomarkers that are directly or indirectly associated with the regulation of energy homeostasis. Among the plasma biomarkers related to energy intake, the concentrations of some were reported to differ between obese and normal weight dogs, such as insulin, ghrelin, leptin and adiponectin [10–13]. Obesity in dogs also results in low-grade systemic inflammation which may contribute to the development of metabolic disorders [7].

Given the health issues related to obesity and the physiology of energy balance, it would be a better strategy to prevent the development of excessive fat stores than to manage established excess weight through weight-loss programs. Numerous investigations in various countries have identified risk factors that were associated with excess weight in overweight or obese but otherwise healthy dogs. The main reported risk factors are neutering, especially in females, high feeding frequency, sedentary lifestyle and specific breeds or cross-breeds [2, 14–16]. Human studies have determined factors during the gestational and infancy periods that affect the degree of obesity in adulthood. It was demonstrated that an obese mother or high gestational weight gain may lead to an elevated body-mass index (BMI) in the offspring [17]. A high birth weight and weight gain during growth have also been identified as risk factors for overweight in adulthood for humans [17] and cats [18].

Although early risk factors have been widely studied in humans, little work has been conducted in small animal medicine to identify early growth patterns correlated with risks of becoming overweight in adulthood. Moreover, and to the authors’ knowledge, an association between the biomarkers mentioned above and the development of obesity in adulthood has not been studied in dogs.

The aims of our study were (i) to show that excess weight gain without an obvious underlying cause could occur between dogs of the same age, sex, sexual status and breed and raised on the same diet in the same environment and (ii) to identify early predictive factors such as parental and neonatal characteristics, energy intake or expenditure, or plasma biomarkers, associated with overweight or obesity in adulthood.

## Methods

### Animals

The current investigation involved 24 female Beagle dogs, which were studied from birth to 24 months of age. We expected to have groups that present a significant difference of FM% between two grades of BCS. Assuming that the difference of FM% between two grades of BCS is approximately 5% [19], the sample size was established considering 3 pairwise comparisons (corresponding to BCS 5, 6 and 7) of FM% means with a first type error of 0.5 and a power of 0.8. The maximum sample size per group obtained was 7.47, therefore 8 dogs were allocated to each of the three groups [20]. Furthermore, the sample size of 24 dogs was suitable for both the capacity of Oniris’ facilities to guarantee animal welfare and to ensure that the sampling workload could be conducted in reliable conditions by one person in order to avoid manipulation bias.

They were the offspring of 10 litters (10 mothers and 7 fathers), were housed by litter in the same breeding centre (Isoquimen SL., Barcelona, Spain), weaned at 10 weeks, and neutered at the age of 8 months.

All dogs received an annual veterinary check-up and were vaccinated against canine distemper, canine adenovirus type 2, canine parainfluenza virus, canine parvovirus, rabies, and received worming treatments, at 2.5, 4, 12, and 22 months of age. A registered veterinarian was available to carry out additional veterinary treatments if required, and had the authority to withdraw dogs from the study if any adverse events occurred. Two dogs (one at 12 and one at 14 months of age) reached a BCS of 8/9 prior to the end of the study. Then, they were rationed in order to maintain a stable body weight until study completion.

### Housing & diet

Throughout the study the dogs lived in the same environment and were fed in the same manner. All diets were supplied by Royal Canin (Royal Canin SAS, Aimargues, France). Prior to weaning (at 10 weeks of age), puppies were housed by litter with their mother in the breeding centre of Isoquimen SL, and had free access to mother’s milk and dry diet. This dry diet, Medium Starter (protein = 30%DM, fat = 22%DM, 4010 kcal/kg or 16.8 MJ/kg) was available *ad libitum* for both mothers and puppies, making an accurate assessment of the puppies’ nutrition source (dry diet vs maternal milk) impossible.

After weaning, dogs were relocated to Oniris (Nantes, France) and were housed in pairs. Each pair was housed in an outdoor enclosure of 4 m^2^ that included a sheltered place to sleep. Each dog was fed individually *ad libitum* for 3.5 h per day whilst the partner was temporarily removed from the enclosure. From weaning to 10.5 months of age, the dogs were given a dry diet formulated to meet growth requirements, Pediatric Junior Dog (protein =29%DM, fat = 20%DM, 3900 kcal/kg or 16.3 MJ/kg). From 10.5 months of age, in order to avoid too much excess weight gain after spaying, the dogs were fed with a moderate calorie dry maintenance diet (Neutered Adult; protein = 28%DM, fat = 11%DM, 3260 kcal/kg or 13.6 MJ/kg).

Individual food intake (g/day) was recorded daily (except on weekends) from weaning to 24 months of age, on the same calibrated electronic weigh scale (Ohaus Europe, Greifensee, Switzerland; accurate to within 0.2 g). The energy intake was corrected for metabolic body weight (EI; kcal/BW^0.75^) or for fat-free mass (EI_FFM;_ kcal/FFM) which was calculated as follows:

1 $$ {EI}_{FFM}\ \left[{kcal.FFM}^{- 1}\right] = \frac{food\  intake\ \left[{kg.d}^{-\mathrm{1}}\right]\times energy\  content\left[{kcal.kg}^{-\mathrm{1}}\right]}{FFM\left[ kg\right]} $$

Dogs were walked on a leash for at least 15 min twice a week and had access to 1 h/day of free time in a closed garden of 400 m^2^ enriched with agility equipment.

Dogs had free access to water throughout the study.

### Biometric assessment

Early-life data were provided by the breeding centre, including age and BW of parents at mating, parity, weight gain during gestation, litter size and BW of each puppy at birth.

Prior to weaning, puppies were weighed every 2 weeks. Post-weaning, BW was recorded weekly on the same electronic weigh scale (Mettler-Toledo SAS, Viroflay, France; accurate to within 50 g). The withers height was measured at 24 months of age. The morphometric estimation of body fat described by Burkholder and Toll [21] has been validated only on adult dogs, so a the pelvic circumference (PC) and patella-to-calcaneus (PCL) was measured every 2 months from 7 months of age, when dogs had a morphology closer to adult one. The body condition score (BCS) was evaluated monthly from 7 months of age by the same investigator, using a 9-point scale (1 for emaciated, 9 for morbidly obese) as recommended by the WSAVA [22].

The body composition was determined by isotopic dilution (deuterium oxide) at 6, 9, 12, 15 and 24 months of age. Food was withheld for 20 h before and water from 1 h before to 3 h after a subcutaneous tracer injection (physiological saline ^2^H_2_O solution (99.9% 2H/H; Euriso-top, Saint-Aubin, France), 0.5 g/kg), to achieve body water equilibration. Venous blood samples were collected in sterile ethylenediaminetetraacetic acid (EDTA) tubes before and 3 h after injection of the isotope. Total body water was determined in two steps. Firstly, the deuterium enrichment of plasma water was determined by Fourier-transform infrared on a Vector 33-type spectroscope (Brücker SA, Wissembourg, France) as previously described [23]. The deuterium enrichment (2H/H) was used to calculate the dilution space of the isotope, which indicates the total body water content after correction for proton exchanges with non-aqueous molecules [24]. Finally, the fat-free mass (FFM) in dogs was calculated with a canine specific hydration rate [25]:

2 $$ F F M\ \left[ kg\right] = \frac{Total\  body\  water\ \left[ kg\right]}{0.744} $$

The proportion of fat mass (FM%) was calculated as the difference between BW and FFM, divided by BW.

Given that the ideal BW in Beagles should be composed of approximately 80% FFM and 20% FM [21], the ideal weight would be FFM × 1.25. The percentage of excess weight according to estimated ideal weight at 24 months of age was calculated as follows:

3 $$ Excess\  weight\ \left[\%\right]=\frac{BW\ \left[ kg\right]-\left( FFM\ \left[ kg\right] \times 1.25\right)}{FFM\ \left[ kg\right]\times 1.25} \times 100 $$

### Blood sampling

Blood samples were taken from dogs after 20 h of food deprivation, every 2 months from 3 to 17 months of age in order to measure plasma levels of glucose, appetite-related hormones (insulin, ghrelin, leptin and insulin-like growth factor 1 [IGF-1]), and stress markers (cortisol and prolactin).

Additional blood samples were taken every 2 months until 9 months of age, and subsequently every 3 months until 15 months, in order to measure levels of markers of inflammation (C-reactive protein [CRP], adiponectin, interleukin [IL-] 6, IL-8, IL-10 and tumour necrosis factor alpha [TNFα]).

At 7 and 13 months of age, in order to follow the post-prandial plasma kinetics of glucose, insulin, ghrelin and peptide YY_3–36_ (PYY), blood was collected immediately before a meal, and then 15, 30, 60, 90, 120 and 150 min after the meal. To avoid the influence of meal size and eating duration [26], dogs were given 10 min of access to a meal of their standard diet providing 130 kcal/kg metabolic BW (BW^0.75^) or 544 kJ/BW^0.75^ according to the recommendations for kennel dogs [27].

In all cases, blood was collected in heparin-coated sterile vacutainers. Plasma was separated by centrifugation at 5000 *g* for 10 min, then aliquoted and stored at –20 °C in sealed vials until analyses were completed.

### Assays

Glucose was assayed immediately after collection by AlphaTRACK 2, a validated portable canine blood glucose meter (Abbott Animal Health, Abbott Park, IL, USA) using capillary or heparin-venous blood [28].

Plasma insulin and insulin-like growth factor 1 (IGF1) were assayed, as previously used in dogs by immunoradiometric assay (IRMA) [29] and radioimmuno assay (RIA) [30] using human kits (Insulin IRMA KIT, Beckman Coulter, Nyon, Swiss; IGF-1 RIA-CT, Mediagnost, Reutlingen, Germany). Active ghrelin concentration were assayed by enzyme-linked immunosorbent assay (ELISA), using a human kit (Human Acylated Ghrelin Express ELISA, BioVendor, Brno, Czech Republic, validated in dogs [31]). The total PYY and leptin concentrations were assayed by a human PYY ELISA kit and a canine leptin ELISA kit, respectively (Millipore, St. Charles, MO, USA). Cortisol concentration was assayed by a cortisol human RIA kit (Demeditec, Kiel, Germany) which was internally validated (coefficients of variation on 3 levels: A: 60 nmol; B: 200 nmol and C: 550 nmol; inter-assay A: 5%, B: 8%, C: 5%; intra-assay A: 12%, B: 8%, C: 14%). Prolactin concentrations were assayed by a canine prolactin ELISA kit, (Demeditec, Kiel, Germany).

Plasma CRP concentrations were measured using a specific solid phase sandwich immunoassay (Canine C-reactive Protein Assay, Tridelta Development Limited, County Kildare, Ireland). Adiponectin levels were determined using a high-sensitive human adiponectin ELISA kit (Human Adiponectin ELISA High sensitivity, BioVendor, Brno, Czech Republic; validated in dogs [32]). Plasma concentrations of canine IL-6, IL-8 and IL-10 and TNFα were assayed by specific ELISA kits (Quantikine ELISA Canine IL-6, IL-8, IL-10, TNFα, R&D Systems Inc., Minneapolis, MN, USA).

Plasma insulin to glucose concentrations ratio (I:G) was calculated in the unfed and postprandial state as follows:

4 $$ I: G=\frac{insulin\left[\mu U. m{L}^{-1}\right]}{fastingglucose\left[ mg. d{L}^{-1}\right]} $$

### Energy expenditure assessment

Energy expenditure was determined by indirect calorimetry at 4, 7, 10 and 16 months of age, as validated in dogs by Pouteau et al. [33], with the following minor modifications. Food was withheld for 20 h, after which dogs were placed in a metabolic chamber (60 × 66.5 × 65 cm) for 4 h. The chamber was connected to a breath gas-exchange monitor (Quark RMR, Cosmed, Rome, Italy), which was calibrated at the start and then hourly, using a standard gas mixture. The system was an open-circuit ventilated by atmospheric air, pumped through the metabolism chamber at a flow rate of approximately 8 L/min adjusted for each dog at each age. The rate of flow of CO_2_ production and O_2_ consumption was measured every 5 s. The energy expenditure (kcal/d) was calculated using the abbreviated Weir formula [34]:

5 $$ Energy\  expenditure\ \left[ kcal.{d}^{-1}\right]=\left(1.11\kern0.75em \times \kern0.5em  r C{O}_2\left[ L.{d}^{-1}\right]+3.94\kern0.75em \times \kern0.5em  r{O}_2\left[ L.{d}^{-1}\right]\right) $$

After an approximately 40-min equilibration period, the energy expenditure was averaged on rolling 20-min periods. The resting energy expenditure (REE) was assumed to correspond to the lowest rolling mean of the energy expenditure during the 4 h of measurement, when the dog was calm but not asleep.

The REE was corrected (i) for metabolic BW (BW^0.75^) at 4 and 7 months and expressed as REE (kcal/BW^0.75^) and (ii) for FFM at 7, 10 and 16 months and expressed as REE_FFM_ (kcal/FFM), both determinations being performed within a window of 30 days. The activity level was not measured.

### Data analysis

All statistical analyses were performed using R software (R Foundation for Statistical Computing, Vienna, Austria) [35]. Graphs were prepared using GraphPad Prism software (GraphPad Software Inc., San Diego, CA, USA).

In order to distinguish dogs in three distinct groups, a principal component analysis (PCA) was performed on quantitative and active variables: FM%, FFM and PC, on dogs aged of 24 months. This was followed by a hierarchical clustering classification (HCPC) which was realized using Ward algorithm with euclidean distance on the two first principal components. The three identified groups were named IW, OW1 and OW2. The BCS is a qualitative variable, so it was included as supplementary qualitative variable in the PCA.

In order to assess the impact of parental and gestational factors on FM%, FFM and PC, and parental characteristics were included as supplementary variables in the PCA.

In order to determine the age at which groups became significantly different, additional PCAs were conducted on FM%, FFM and PC at the ages of 6, 9, 12 and 15 months. Confidence ellipses (95% confidence level) were constructed for the three groups in each PCA.

Mean and standard deviation (SD) were computed for each variable at 24 months of age in the three groups. The independence between the groups and the parents was assessed by Fisher’s exact test.

In order to characterize the growth throughout the study period, individual growth curves, defined as BW over time were plotted. Growth curves usually show a sigmoid profile and are fitted by a Gompertz function [36]:

6 $$ B{W}_t= B{W}_{max}\times {e}^{-\alpha \times {e}^{- kt}} $$

where:

t is age in weeks,

BW_t_ is body weight at time t in kg,

BW_max_ is the maximum body weight, also named mature body weight,

α is an expression of the ratio between mature and birth body weight (BW_b_; $\alpha = ln\left(\frac{B{ W}_{max}}{B{ W}_b}\right)$)

k is the maturation rate, which corresponds to the velocity to reach the adult body weight and e is Euler number.

The growth period could be divided into two periods by the point of inflection: the first period corresponding to an increasing growth rate and the second period to a decreasing growth rate. The maximum of weight velocity, calculated as $\frac{B{ W}_{max}\times k}{e}$ occurs at the point of inflection (PI) of coordinates $\left({t}_{PI}=\frac{ \ln \left(\alpha \right)}{k};\kern0.5em  B{W}_{PI}=\frac{B{W}_{max}}{e}\right)$.

In order to assess the growth parameters (BW_max_, α and k) of each group, the growth data collected from 0 to 18 months of age were fitted by the Gompertz function using nonlinear mixed effect model tools (saemix package in R software) with the dog as random effect.

To compare the evolution of BW of the groups during specific life-stage periods, we split the study duration into five periods. Linear mixed effects models were performed on age, BW, and groups over the following periods: before weaning (0 to 2.5 months of age), pseudo-linear growth (2.5 to 6 months of age), before spaying (6 to 8 months of age) and after spaying (8 to 10.5 months of age). Given that the normal weight in 2-week old neonatal puppies is twice that at birth [37], GR_2W_, % was calculated. We also calculated the relative weight gain after sterilisation (%) to assess the susceptibility to gain weight after the surgery. Post-prandial kinetic data (0–150 min) was transformed into area under the curve (AUC) values based on the difference from baseline. In order to interpret energy intake and expenditure, resting energy balance was calculated by subtracting REE_FFM_ from EI_FFM_.

To take into account the repeated measurements for individual dogs in time, linear mixed effects models (nlme package in R software) were performed with dogs as random term.

These models enabled us to assess

i) the interactions between groups and age on BW, FFM, FM%, PC, energy intake and expenditure or on hormonal concentrations,
ii) the correlation between parental factors and biometric data at 24 months of age,
iii) the correlation between the growth rate in the first 2 weeks of life (GR_2W_) and either groups or FM% at 24 months of age.

Independency and normal distribution for residuals and random effects were checked by graphical tools as described in the theory of mixed models effects [38].

Logistic regression analysis was performed on the three groups by pairs (IW–OW1; OW1–OW2, IW–OW2) to attempt to discriminate the groups by

i) parental and gestational variables,
ii) GR_2W_,
iii) BCS, FM% or FFM prior to 2 years of age.

For each identified discriminant factor, a discriminant value was deduced from logistic regression results and was used as a cut-off to differentiate the groups. The goodness of fit was explored through an analysis of deviance table.

In each model, multiple comparisons were taken into account and adjusted *p*-values were calculated by single-step method, proposed in “multcomp” package of R software. All *p*-values were compared to α = 0.05 to establish significant differences.

## Results

All dogs remained healthy throughout the duration of the study.

### Constitution of three groups based on biometric data at 24 months of age

**Fig. 1:**
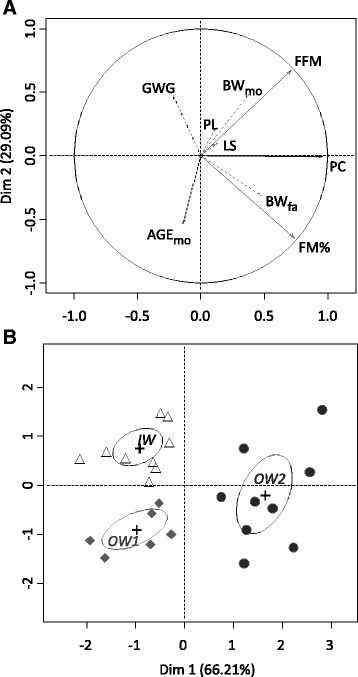
Outputs of the PCA followed by the hierarchical clustering on principal components (HCPC). The analyses were assessed on 24 female Beagle dogs aged 24 months. Groups were described as ideal weight (IW, *n* = 9), slightly overweight (OW1, *n* = 6) and overweight (OW2, *n* = 9). **a**: Factor map of principal and supplementary variables. The principal variables (*solid lines*) are fat-free mass (FFM, kg), fat mass proportion (FM%,) and pelvic circumference (PC, cm). Supplementary variables (*dotted lines*) are fathers’ body weight (BWfa, kg), mothers’ body weight at mating (BWmo, kg,), mothers’ age at mating (AGEmo), previous litters (PL), gestational weight gain (GWG) and litter size (LS). **b**: Confidence ellipses (95% confidence level) around the groups identified by HCPC

In order to categorize dogs according to their status of fatness, a PCA was performed on FM%, FFM (kg) and PC (cm) at 24 month of age. The two first axes explained 95.3% of the total variation (Fig. 1a). A hierarchical clustering of principal components revealed three well separated groups (Fig. 1b), which could be described as dogs with (optimal) ideal weight (IW, *n* = 9), slightly overweight (OW1, *n* = 6) and overweight (OW2, *n* = 9).

**Fig. 2:**
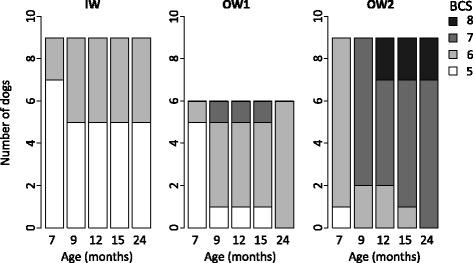
Number of dogs at each BCS at considered ages. Groups were described as ideal weight (IW, *n* = 9), slightly overweight (OW1, *n* = 6) and overweight (OW2, *n* = 9)

The distribution of BCS in the groups at 24 months (Fig. 2) was in line with the groups’ characteristics (Table 1). The OW2 group had significantly higher values of BW, FM%, PC (all *p* < 0.001) and height to withers (*p* = 0.037) than the IW group, although the FFM of the two groups was similar (*p* = 0.25). The OW2 group also had significantly higher values of BW, height to withers, FFM, FM% and PC compared to the OW1 group (*p* < 0.001, *p* = 0.003, *p* < 0.001, *p* = 0.02, *p* < 0.001, respectively).

**Table 1:** Morphometric values of the 24 female Beagle dogs

	6 months						24 months					
	IW		OW1		OW2		IW		OW1		OW2	
Body weight (kg, BW)	10.19	(0.73) **a**	8.84	(1.08) **b**	11.23	(1.25) **c**	13.64	(1.10) **a**	12.44	(1.87) **a**	17.77	(2.18) **b**
Fat-free mass (kg, FFM)	8.63	(0.74) **a**	7.26	(0.76) **b**	8.96	(0.96) **a**	10.10	(0.71) **a**	8.38	(0.97) **b**	10.98	(1.54) **a**
Fat mass (%, FM%)	18.44	(2.93) **a**	19.23	(1.99) **a**	21.11	(3.23) **a**	26.13	(3.11) **a**	32.90	(2.08) **b**	37.39	(3.23) **c**
Pelvic circumference (cm, PC)	47.21	(2.02) **a**	46.25	(3.93) **a**	47.00	(3.97) **a**	44.27	(2.69) **a**	43.52	(1.78) **a**	53.10	(1.99) **b**
Excess body weight (%)							7.3	(3.6) **a**	15.3	(5.0) **b**	22.8	(3.8) **c**

Values are expressed as mean (standard deviation). The letters identify significant differences (*p* <0.05) between groups difference within the same parameter. The dogs were separated into 3 groups using a hierarchical classification on principal components on FM%, FFM and PC. Groups were described as ideal weight (IW, *n* = 9), slightly overweight (OW1, *n* = 6) and overweight (OW2, *n* = 9)

The OW1 and IW groups had similar BW and height to withers at 24 months of age (*p* = 0.208, *p* = 0.201, respectively), while there was a significant difference in FFM and FM% between groups (*p* = 0.010, *p* < 0.001, respectively). The mean percentage of excess weight as calculated by Eq. 3 was significantly higher in the OW2 than in the IW (*p* < 0.001) and OW1 group (*p* = 0.002), and higher in the OW1 than in the IW group (*p* = 0.001).

### Parental and gestational factors

Parental characteristics, such as BW and age of parents at mating, parity, gestational weight gain and litter size were added as supplementary variables to the PCA assessed in 24 months old dogs (Fig. 1). A linear regression model confirmed that the BW of the mothers was significantly and positively correlated with FFM of their offspring at 24 months (*p* < 0.001) and the BW of the fathers was significantly and positively correlated with FM% of their offspring at 24 months (*p* = 0.002). Logistic regression analysis did not identify any parental characteristics differentiating the groups.

### Weight gain curves

Table 2 characterizes the fitted growth curves for each group. The OW2 group was found to be significantly different from the IW group in many respects, with significantly increased values of BW_max_, t_PI_ and BW_PI_ (all, *p* < 0.05). Both overweight groups (OW1 and OW2) exhibited a lower value for the constant k than the IW group (both, *p* < 0.05). The OW1 group also appeared quite different to other groups, with significantly lower values for BW_max_, BW_PI_ and maximum of weight velocity (all, *p* < 0.05).

**Table 2:** Parameters of fitted weight curves and 95% confidence intervals

	IW			OW1			OW2		
	Mean	(SD)	95% CI	Mean	(SD)	95% CI	Mean	(SD)	95% CI
BW_max_, maximum body weight (kg)	13.10	(0.13) **a**	[12.81; 13.39]	12.03	(0.11) **b**	[11.75; 12.30]	16.16	(0.16) **c**	[15.79; 16.54]
α, mature:birth body weight ratio	2.55	(0.16) **a**	[2.18; 2.92]	2.50	(0.14) **a**	[2.15; 2.85]	2.60	(0.18) **a**	[2.19; 3.01]
k, maturation rate	0.088	(0.005) **a**	[0.077; 0.099]	0.078	(0.004) **b**	[0.069; 0.087]	0.075	(0.005) **b**	[0.065; 0.085]
t_PI_ (weeks)	10.64	(0.22) **a**	[10.15; 10.81]	11.73	(0.26) **b**	[11.06; 12.03]	12.73	(0.27) **b**	[12.12; 12.90]
BW_PI_ (kg)	4.82	(0.11) **a**	[4.71; 4.93]	4.42	(0.10) **b**	[4.32; 4.52]	5.95	(0.14) **c**	[5.08; 6.08]
Maximum of weight velocity	0.42	(0.06) **a**	[0.36; 0.49]	0.35	(0.048) **b**	[0.30; 0.39]	0.45	(0.07) **a**	[0.38; 0.52]

Values are expressed as mean (standard deviation). Letters represent a significant difference (*p* <0.05). Groups were described as ideal weight (IW, *n* = 9), slightly overweight (OW1, *n* = 6) and overweight (OW2, *n* = 9). Details regarding the fitted curves can be found in the Materials and Methods

### Life-stage periods of weight gain

#### Birth to weaning

At birth, BW_b_ did not significantly differ between groups, with IW, OW1 and OW2 groups having an average BW_b_ (SD) of 0.44 (0.07) kg, 0.47 (0.14) kg and 0.39 (0.06) kg, respectively.

**Fig. 3:**
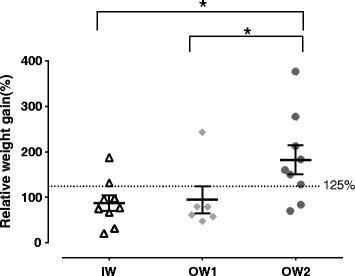
Scatter plot of GR2W (%) of three groups, during the two first weeks of life. Groups were described as ideal weight (IW, *n* = 9), slightly overweight (OW1, *n* = 6) and overweight (OW2, *n* = 9). Data are presented by groups, the line represents the median of the group; error bars represent the interquartile range. * significant difference (*p* < 0.05) between groups as identified by a linear regression model

The GR_2W_ (SD) was significantly higher in the OW2 (182.4% (30.2)) than in IW (87.1% (50.5), *p* = 0.015) and OW1 groups (94.4% (73.8), *p* = 0.040) (Fig. 3). In logistic regression GR_2W_ discriminated the OW2 from the IW group, by being close to statistical significance (*p* = 0.051). A threshold of GR_2W_ of 125% was calculated to discriminate groups. Moreover, among the 10 dogs with GR_2W_ higher than 125%, 7 belonged to the OW2 group, 2 to IW and 1 to OW1. GR_2W_ was also significantly correlated with FM% at 24 months (*p* = 0.038), independent of group allocation.

**Fig. 4:**
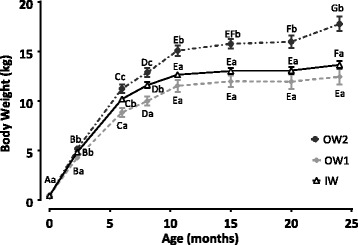
Body weight as a function of time in 24 female Beagle dogs. Groups were described as ideal weight (IW, *n* = 9), slightly overweight (OW1, *n* = 6) and overweight (OW2, *n* = 9). Values represent means of groups, while error bars represent standard error of the mean. Upper-case letters identify significant differences (*p* < 0.05) within a group; lower-case letters identify differences (*p* < 0.05) between groups

From 6 to 24 months of age, the BW of the OW2 group was significantly greater than those of the other groups (*p* < 0.05). Between 12 and 24 months, the BW of the IW did not significantly differ from the OW1 groups (Fig. 4).

We also compared the evolution of BW and weight gain during 3 growth periods (2.5 to 6 months, 6 to 8 months and from 8 to 10.5 months of age). At weaning (2.5 months), the BW of the OW2 and IW groups were significantly greater than those of the OW1 group (*p* = 0.003 and *p* = 0.041, respectively; Fig. 4). Body weight increased significantly more in the OW2 group than in the IW for all three periods concerned (all, *p* < 0.001). The rate of weight gain (SD) over the 8 to 10.5 month period was higher in the OW2 (14.93% (6.66)) compared to the IW group (8.53% (3.70); *p* = 0.016); the OW1 group (12.22% (3.18)) was not significantly differ from the IW (*p* = 0.106) and OW2 (*p* = 0.517) groups.

### Determination of the minimum age at which groups are distinct

According to the PCA and HCPC processed at 6, 9, 12 and 15 months, significant differences between groups were established in accordance with the confidence ellipses (95% confidence level). In dogs aged 6 months, the OW2 group was significantly distinct from the IW and the OW1 groups while there was some overlap between the IW and the OW1 groups. From 9 months of age, all groups were significantly distinct.

The results of PCA and HCPC for determination of the earliest age of differentiation between groups were supported by an analysis of the biometric data (BCS, FM%, FFM, and PC) over the 6 to 24 month period. The distribution of BCS values remained relatively stable over time in the IW group but in the OW1 and the OW2 groups, BCS values increased (Fig. 2). At 7 months of age, the BCS discriminated OW2 from both the IW (*p* = 0.012) and OW1 (*p* = 0.016) groups. Among the 11 dogs with a BCS of 6 at 7 months, 8 belonged to the OW2 group, 2 to the IW and 1 to the OW1 group. From 9 months onwards, the OW2 group had significantly greater FM% than the IW group (*p* = 0.005 at 9 months); from 12 months onwards, the FM% of the OW2 group was higher than the OW1 group (*p* = 0.029 at 12 months). While the FFM of the OW1 group was always significantly inferior to the other groups (both *p* < 0.014), no significant difference was observed between the FFM values of the IW and OW2 groups whatever the age (*p* = 0.18). Finally, the PC of the OW2 group was significantly greater from 9 months of age onwards compared to the other groups (both *p* < 0.001 at 9 months).

### Energy intake and expenditure

**Fig. 5:**
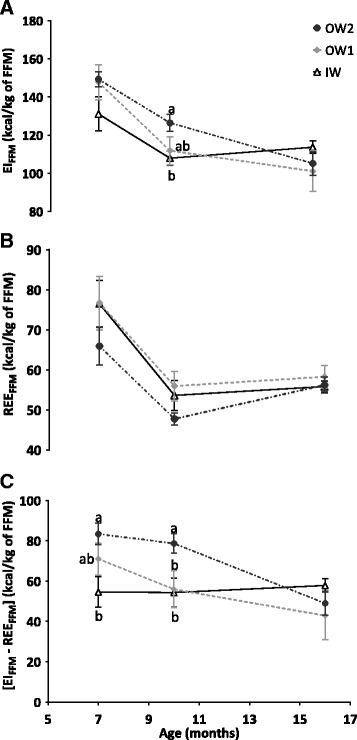
Evolution of: **a**: energy intake (EI); **b**: resting energy expenditure (REE) and (**c**): EI less REE. All the values were adjusted for fat-free mass (FFM) in 24 female Beagle dogs. Groups were described as ideal weight (IW, *n* = 9), slightly overweight (OW1, *n* = 6) and overweight (OW2, *n* = 9). Values represent means of groups, while error bars represent standard error of the mean. Letters identify significant differences (*p* < 0.05) between groups at the same age

Energy intake and expenditure were analysed over three age periods: 4 to 7 months (adjusted for metabolic BW), 7 to 10 months and 10 to 16 months (each adjusted for FFM, Fig. 5). One dog from the group IW was not included into the analysis, the measurements were not reliable. Actually, the dog had difficulties to remain calm all along the experimental procedure.

At 4 months, all the groups had a similar EI and REE. Over 4 to 7 months, EI decreased significantly (*p* < 0.0001) and similarly in all groups, whereas for REE, there was a group-age interaction (*p* = 0.007), with an increase observed in the IW group and a decrease observed in the OW2 group.

From 7 to 10 months of age, both EI_FFM_ and REE_FFM_ decreased significantly (*p* < 0.001) and similarly in all groups. At 10 months, EI_FFM_ was significantly higher in the OW2 group than in the IW group (*p* = 0.013), while the REE_FFM_ did not significantly differ (Fig. 5a). On considering the resting energy balance, [EI_FFM_ – REE_FFM_] over the 7 to 10 months period, values for the OW2 group were significantly higher than the IW (*p* = 0.001) and the OW1 group (*p* = 0.035) irrespective of time (Fig. 5c). However no significant changes were observed for any of the groups over time.

### Hormonal variations

Prior to 11 months of age, no differences were found in basal leptin concentration between the groups. From 11 months, however, the leptin level was significantly higher in the OW2 group than in the IW group irrespective of age (*p* = 0.010). The basal plasma IGF1 concentration decreased significantly in all groups, until it reached a plateau at 13 months of age (*p* < 0.001). Before 11 months of age, the basal plasma IGF1 was lower in the OW1 group than in the IW and the OW2 groups (*p* = 0.045 and *p* = 0.061, respectively). From 11 months of age, the level of IGF1 did not significantly differ among groups. The groups did not significantly differ by their I:G ratio, adiponectin, ghrelin, cortisol, prolactin, CRP, IL-8 and IL-10 measured in the fasting state (Additional file 1).

Statistical analyses for IL-6 and TNFα were not performed, as the majority of samples were below the level of detection.

**Fig. 6:**
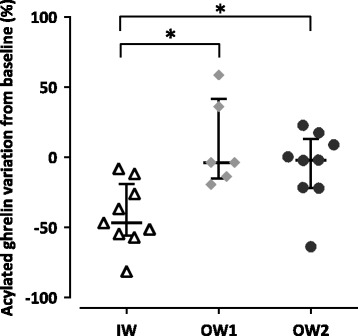
Baseline-adjusted variations of acylated blood ghrelin 60 min after a test meal. Values were obtained from 24 female Beagle dogs at the age of 7 months. Groups were described as ideal weight (IW, *n* = 9), slightly overweight (OW1, *n* = 6) and overweight (OW2, *n* = 9). Data are presented by groups, the line represents the median of the group; error bars represent the interquartile range. Lower-case letters identify significant differences (*p* < 0.05) between groups

Analysis of post-prandial kinetic data showed that before 60 min, the baseline-adjusted acylated ghrelin values were significantly lower in the IW group than in the OW1 group (*p* = 0.027). At 60 min after the test meal, the variation of acylated ghrelin compared to baseline was lower in the IW group than in the OW1 (*p* = 0.002) and the OW2 groups (*p* = 0.011) (Fig. 6). After 60 min, the baseline-adjusted acylated ghrelin values did not differ between groups. No significant difference were detected between groups concerning whether the AUC values of ghrelin, PYY, plasma glucose, insulin or postprandial I:G ratio (Additional file 2), or variations from baseline (excepted for ghrelin).

## Discussion

This is, to our knowledge, the first longitudinal study in growing dogs to investigate predictive factors that could explain becoming overweight and obese in adulthood.

The BCS values of the dogs aged 24 months ranged from 5 to 8, thereby confirming that some dogs can be more susceptible to gain body fat than others of the same breed, fed the same diet and housed under the same environmental conditions. FM%, FFM and PC at 24 months of age retrospectively identified three well distinct groups of dogs that differed by their median BCS. When categorized in this manner, *a posteriori*, 62.3% of dogs were classified as having become overweight (Table 1). This is consistent with the reported prevalence of overweight dogs worldwide [2, 3, 15, 39].

A number of parental and neonatal parameters were examined to determine early predictive markers of overweight status in adulthood. Fat-free mass and fat mass of the offspring at 24 months of age were positively correlated with the BW of the mother and the father, respectively, but parental BWs were insufficient to clearly discriminate IW, OW1 and OW2 groups. This pilot study was conducted on 24 dogs born from 7 fathers and 10 mothers, which is a small sample size. The Fisher’s exact test showed that despite this lack of power, the parental contribution should not be removed from future investigations.

In contrast to human studies, BW at birth did not correlate to the overweight status, FFM at adulthood [40, 41] or parental BW [42]. The earliest predictive marker of adult overweight status in our study was GR_2W_. Seven out of the ten dogs with a GR_2w_ greater than 125% (cut-off point determined by the regression logistic model, see Data analysis section) belonged to the OW2 group. Despite the lack of strictly comparable data (% weight gain over the first 2 weeks of life) in humans, our finding is similar to those in human studies, which have shown that high weight gain in early life stages is associated with the development of adult obesity [43–45]. Neonatal growth will be impacted by the availability and composition of the dams’ milk, which depend on litter size. We were unable to see a clear influence of litter size on GR_2W_, and the explanation for GR_2W_ as a predictive factor for overweight status remains unclear. Future investigations with larger populations are needed to establish the impact of the dam’s milk and/or litter size on the overweight status of offspring.

Comparisons of weight gain between groups were performed by Gompertz-fitted curves over 18 months. The OW2 group presented the highest BW_max_ and the lowest k (maturation rate) compared to the IW group (Table 2). Given that α was the same in all dogs as a breed-size characteristic, this indicates a delayed maximum weight velocity (Table 2, t_PI_ IW < t_PI_ OW2); human studies have reported contrasting findings on the association of weight velocity and age of PI, with the risk of becoming overweight [46]. Maximum weight velocity was similar in the IW and OW2 groups, but lower in the OW1 group, which could correspond with the higher FFM in the IW and OW2 groups. Increased frequency of measurements of morphometric parameters could help to clarify the underlying correlation of weight gain patterns with the overweight status in adulthood, as has been suggested in humans [46, 47]. Analysis of BW change by growth period confirmed the approach by the Gompertz model: the groups displayed a similar pseudo-linear growth followed by a deceleration of growth, which was both delayed and reduced in the OW2 group compared to the IW group. After spaying but before the change of diet, BW increased in all groups but the rate of weight gain was higher in the OW2 (~15%) compared to the IW group (~8%). This suggests that spaying could aggravate excessive weight gain in dogs predisposed to obesity. Excessive fat-mass deposit was reflected in a BCS > 5/9 in 8/9 dogs in the OW2 group at 7 months of age. This is consistent with previous studies, either in cats, where lean and overweight phenotypes could be identified as early as 8 months according to the BCS [48], or in humans, where it was demonstrated that overweight status in childhood or adolescence is generally maintained or increased in adulthood [49]. The 9 point-scale BCS, although currently only used in adult dogs, may help veterinarians quickly and easily identify adolescent dogs (from 6 months of age) that may be at risk of becoming overweight in adulthood. Without an external intervention to regulate energy intake, the dogs in the OW2 group gained excess fat mass from 6 months of age. The establishment of growth charts, which take into account the size and body weight growth similar to the WHO’s growth charts would be an interesting tool to monitor growth in puppies.

In healthy dogs, excess weight gain is due to an energy imbalance. In this pilot study, the energy balance was approximated by the analysis of the energy intake and the REE.

The fact that both EI and REE corrected for metabolic BW did not differ between the groups at 4 months could suggest that either the OW2 group had no energy imbalance at that stage in life or that the variation within groups was too large to detect a difference. During the 7 to 10 month period, in which spaying occurred, we found that EI_FFM_ and REE_FFM_ decreased by approximatively 18 and 25%, respectively (Fig. 5a, b) in the three groups. These decreases could be related to spaying and/or the end of growth. The observed decrease in REE is consistent with one study in dogs and another study in cats, which suggested that energy requirements decreased by approximately 30% after gonadectomy in both species [50, 51], resulting in a general recommendation to reduce calorie intake after neutering [37]. Over this 7 to 10 month period, [EI_FFM_–REE_FFM_] (Fig. 5c) was higher in the OW2 group compared to the IW group which would explain the increase in fat mass in the OW2 group. Although the dogs were subject to the same environmental conditions, an individual measurement of physical activity could have helped to understand the link between the excess weight gain and the differences between EI_FFM_ and REE_FFM_. Although time-restricted feeding could have impacted overall weight gain [52], all dogs were in same conditions, and thus have limited the impact of this outcome on our study.

Thus, it seems that the OW2 group had a poorer control of energy balance regulation. This could be linked to the basal level of leptin in this group compared to the others. At 7 months, the leptin levels did not differ between the IW and OW2 groups which could be considered normal as the FM% of these groups were not significantly different at 6 months. After gonadectomy, the leptin level was significantly higher in the OW2 than in the IW group, which is in line with previous studies in dogs which correlated the level of basal leptin to BCS and FM% of dogs regardless of age, breed and sex [53]. This study failed to show plasma leptin as an early marker of excess weight gain in later life, indicating that leptin’s most important role is the preservation of existing body fat stores [54].

We also found that acylated ghrelin levels decreased more rapidly and were significantly lower 60 min after a test meal in the IW group compared to the OW1 and OW2 groups when measured at 7 months of age. A similar observation has been made in obese cats [55]. Our findings suggest that the delayed suppression of acylated ghrelin in the OW2 and OW1 groups might impact their short-term regulation of energy balance [56] by facilitating overfeeding.

More sensitive methods for determination of plasma biomarkers might also help to detect earlier differences between groups. Our results warrant further investigations on energy expenditure during growth and following neutering in a larger group of dogs in order to limit the impact of individual variability [57]. Further investigations might also include differences in gastrointestinal microbiota [58, 59] and variations in gene expression in adipose tissue [60].

## Conclusion

Albeit small, the sample size used in the current study was sufficient to highlight differences in the development of overweight and obesity between dogs matched for age, sex and breed and raised under the same conditions. Among the predictive factors of adult obesity which were identified, the neonatal growth rate and adolescent BCS could be exploited in a clinical setting. Neonatal growth rate might help breeders identify dogs that should be dietary restricted from an early age. Adolescent BCS values might help veterinarians deliver specific nutritional advice for dogs at higher risk of becoming overweight before neutering. Any practical recommendations, however, are contingent upon validation of our findings in larger populations and in different breeds and sexes.

## Additional files

Additional file 1: Hormonal values by group and by age. Values are expressed as mean (standard deviation). (CSV 3 kb)

Additional file 2: Averaged AUCs at 7 months of age during a 150 min postprandial kinetic. Values are expressed as mean (standard deviation). Their comparison was assessed by a linear regression model analysis. I:G correspond to the ratio between plasma insulin and glucose. Letters a and b represent significant differences (*p* < 0.05). (CSV 457 kb)

## Abbreviations

AUC: Area under the curve
BCS: Body condition score
BW: Body weight
BW_b_: Body weight at birth
BW_max_: Maximal body weight in the Gompertz model
EI: Energy intake corrected for BW^0.75^
EI_FFM_: Energy intake corrected for FFM
FFM: Fat free mass
FM%: Percentage of fat mass
GR_2W_: Growth rate in the 2 first weeks of life
HCPC: Hierarchical classification on principal components
I:G: Plasma insulin to glucose concentrations ratio
PC: Pelvic circumference
PCA: Principal components analysis
PYY: Peptide tyrosine-tyrosine
REE: Resting energy expenditure corrected for BW^0.75^
REE_FFM_: Resting energy expenditure corrected for FFM
